# Potential Involvement of *MnCYP710A11* in *Botrytis cinerea* Resistance in *Arabidopsis thaliana* and *Morus notabilis*

**DOI:** 10.3390/genes15070853

**Published:** 2024-06-28

**Authors:** Hui An, Donghao Wang, Lin Yu, Hongshun Wu, Yue Qin, Shihao Zhang, Xianling Ji, Youchao Xin, Xiaodong Li

**Affiliations:** 1 Guangxi Key Laboratory of Sericulture Ecology and Applied Intelligent Technology, Hechi University, Hechi 546300, China; 06034@hcnu.edu.cn (H.A.); qinyue1161@163.com (Y.Q.); 2021660014@hcnu.edu.cn (S.Z.); 2 Guangxi Collaborative Innovation Center of Modern Sericulture Silk, School of Chemistry and Bioengineering, Hechi University, Hechi 546300, China; 3 College of Forestry, Shandong Agricultural University, Tai’an 271018, China; wangdh1204@163.com (D.W.); 19860915990@163.com (L.Y.); 15578263347@163.com (H.W.); xlji@sdau.edu.cn (X.J.)

**Keywords:** cytochrome P450, *B. cinerea*, mulberry, *MnCYP710A11*

## Abstract

Cytochrome P450 (CYP) is a crucial oxidoreductase enzyme that plays a significant role in plant defense mechanisms. In this study, a specific cytochrome P450 gene (*MnCYP710A11*) was discovered in mulberry (*Morus notabilis*). Bioinformatic analysis and expression pattern analysis were conducted to elucidate the involvement of *MnCYP710A11* in combating *Botrytis cinerea* infection. After the infection of *B. cinerea*, there was a notable increase in the expression of *MnCYP710A11*. *MnCYP710A11* is overexpressed in Arabidopsis and mulberry and strongly reacts to *B. cinerea*. The overexpression of the *MnCYP710A11* gene in Arabidopsis and mulberry led to a substantial enhancement in resistance against *B. cinerea*, elevated catalase (CAT) activity, increased proline content, and reduced malondialdehyde (MDA) levels. At the same time, H_2_O_2_ and O_2_^−^ levels in *MnCYP710A11* transgenic Arabidopsis were decreased, which reduced the damage of ROS accumulation to plants. Furthermore, our research indicates the potential involvement of *MnCYP710A11* in *B. cinerea* resistance through the modulation of other resistance-related genes. These findings establish a crucial foundation for gaining deeper insights into the role of cytochrome P450 in mulberry plants.

## 1. Introduction

Cytochrome P450 (CYP) is one of the most important enzymes in the oxidoreductase family, which uses heme-thiolate as a cofactor [1]. *CYP* functions as a protective agent for plants, defending them against various pathogenic microorganisms and pests by facilitating the synthesis and metabolism of numerous physiologically essential compounds [2,3,4,5,6]. *CYP* operates within an intricate network of plant defense mechanisms [7]. These defense mechanisms encompass hypersensitivity and the suppression of specific plant pathogen growth [8,9]. The infection of *Pseudomonas syringae* triggered a hypersensitive response (HR), leading to an upregulation of the *CYP76C2* gene in *Arabidopsis thaliana*. The activation of the *CYP76C2* gene is associated with injury, senescent cell culture, and leaf senescence [10,11]. *CYP51H10* is responsible for the synthesis of antimicrobial oleanane-triterpene saponins, which confer resistance to root-infecting fungi in oats [12].

*CYP710* is a member of the *CYP* superfamily and is believed to have originated from the *CYP51* family through evolution [13]. The transformation of β-sitosterol into stigmasterol occurs through a singular enzymatic process catalyzed by sterol C-22 desaturase, facilitated by the cytochrome P450 710A11 (CYP710A11) enzyme [14]. As of now, there is limited knowledge regarding the function of *CYP710A11* in the context of *B. cinerea* infection.

Mulberry is an important economic tree. Its leaves are the main feed of silkworm, and the fruit is rich in nutrition, active substances, good taste, and has high edible and medicinal value [15,16,17,18]. In addition, mulberry is also used for ecological control because of its strong resistance to stress [19]. *B. cinerea* is a necrotrophic fungal pathogen capable of infecting a broad spectrum of plant species, including significant agricultural crops [20,21]. Its primary targets are tender tissues like fruits, vegetables, and flowers. This fungus is prevalent globally and is recognized as a highly economically impactful plant pathogen, leading to substantial crop losses pre- and post-harvest [22]. Simultaneously, *B. cinerea* stands as a primary pathogenic fungus affecting mulberry plants [23].

The transcriptomic data detailing the resistance of mulberry (*M. notabilis*) to *B. cinerea* infection provide valuable insights for investigating the resistance of *MnCYP710A11* to *B. cinerea* infection [24]. According to the preceding transcriptome data, we observed a significant increase in *MnCYP710A11* expression levels in *M. notabilis* during *B. cinerea* infection. The resistance conferred by *MnCYP710A11* against *B. cinerea* infection was investigated. In addition, in order to explore the role of *MnCYP710A11*, we induced the overexpression of *MnCYP710A11* in both Arabidopsis and mulberry trees. We examined the resistance of transgenic Arabidopsis and transiently overexpressed mulberry using diverse methods, confirming the involvement of *MnCYP710A11* in the defense response of these transgenic plants. These results offer preliminary insights into the resistance mechanism of *CYP710A11* and have established a basis for further elucidating the function of *CYP710A11*. At the same time, this serves as a reference for investigating *CYP710A11* in other plant species and presents a potential target gene for enhancing mulberry’s resistance to *B. cinerea*.

## 2. Materials and Methods

### 2.1. Plant Materials and Growth Conditions

The genetic background of *A. thaliana* was Columbia-0 (Col-0), which was cultured in a growth chamber at 22 °C, with 60–80% relative humidity and a 12 h day cycle. Mulberry trees were cultured in a growth chamber at 25 °C, with a 12 h day cycle and 75% humidity.

### 2.2. RNA Isolation and Quantitative Real-Time PCR

Plant tissues were processed for total RNA extraction utilizing CTAB-pBIOZOL (Bioer, Hangzhou, China) following the manufacturer’s guidelines. The initial complementary DNA (cDNA) strand was generated using gDNA Eraser (Takara, Kusatsu, Shiga, Japan) in conjunction with the PrimeScript RT reagent Kit (Takara, Kusatsu, Shiga, Japan), subsequently followed by the synthesis of the second cDNA strand. The qRT-PCR analysis was conducted utilizing the SYBR Green PCR Master Mix (Takara, Kusatsu, Shiga, Japan) on both the Step One and Step OnePlus real-time PCR platforms (Applied Biosystems, Waltham, MA, USA). The Actin gene was employed as the internal reference gene. The qRT-PCR analysis was repeated using three different techniques, with the specific qRT-PCR primer sequences detailed in Table S1. The expression levels were analyzed by using the 2^−ΔCT^ method [25].

### 2.3. Bioinformatic Analysis

The *MnCYP710A11* (L484_021687) amino acid sequence was downloaded from a mulberry genome database (https://morus.biodb.org/morusdb/, accessed on 17 May 2023). Different species of CYP710A11 amino acid sequences were obtained from the National Center for Biotechnology Information database (https://www.ncbi.nlm.nih.gov/, accessed on 17 May 2023). ClustalX software (v. 1.83) was used to align amino acid sequences [26]. The phylogenetic tree was built utilizing the adjacency method as implemented in Molecular Evolutionary Genetic Analysis (MEGA 7) software with a bootstrap value of 1000 [27].

### 2.4. Transformation of A. thaliana

The complete coding sequence of the mulberry *MnCYP710A11* gene was inserted into the pLGNL expression vector using *Kpn*I and *EcoR*I restriction enzymes [28]. Subsequently, the modified plasmid was introduced into the *Agrobacterium tumefaciens* LBA4404 strain. The positive *A. tumefaciens* containing *MnCYP710A11* was transformed into *Arabidopsis* by using the flower dip method [29]. The T3 homozygous lines were studied.

### 2.5. Histochemical GUS Staining

Histochemical analysis of GUS activity is a modification of the procedure previously described [30]. First, the obtained homozygous Arabidopsis seeds overexpressing the *MnCYP710A11* gene were sterilized, and then, the sterilized seeds were spread in an MS medium for culturing. After 2–3 days of cultivation, the plants underwent a water rinse followed by immersion in a GUS staining solution comprising 1 mM 5-bromo-4-chloro-3-indolyl-β-d-glucuronic acid (X-Gluc), 50 mM sodium phosphate (pH = 7.0), 1 mM ethylenediaminetetraacetic acid (EDTA), 0.1% Triton X-100, 50 mM potassium ferricyanide, and 50 mM potassium ferrocyanide. The samples were then incubated at 37 °C for 8–16 h. To improve the visibility of GUS staining, chlorophyll was eliminated using 70% (*v*/*v*) ethanol. For GUS staining of mulberry leaves, first, the plant leaves were invaded by Agrobacterium tumefaciae containing the plant expression vector of the GUS fusion gene by using the vacuum immersion method. After co-culturing, the mulberry leaves were immersed in the GUS staining solution for staining, and finally decolorized with 70% (*v*/*v*) ethanol.

### 2.6. Transformation of Mulberry

*A. tumefaciens* LBA4404 harboring the pLGNL-*MnCYP710A11* vector was prepared using a transformation solution (1/2 MS, 5% sucrose, 200 µM acetosyringone, 0.05% Tween-20, pH 5.6) and adjusted to a final OD600 of 0.5. Fifteen-day-old mulberry seedlings were immersed in the LBA4404 transformation solution containing pLGNL-*MnCYP710A11* and subjected to vacuum treatment at room temperature for 20 min [31].

### 2.7. Transgenic Plants Inoculated with B. cinerea

To assess the resistance of transgenic Arabidopsis to *B. cinerea* (MM1 strain), a resistance test was conducted. Transgenic seeds were germinated on 1/2 Murashige and Skoog (MS) agar medium. After 7 days, the seedlings were transplanted into nutrient soil pots and cultivated under conditions of 24 °C/22 °C with a 16 h light and 8 h dark cycle. Mycelium fragments were then applied to the leaves of 21-day-old plants for the experiment. *B. cinerea* was initially cultured on a PDA plate and cultured in an incubator at 25 °C for 2~3 days. The fungus was then harvested from the edge of the *B. cinerea* fungus colony using a sterilized hole punch, and the fungus was inoculated on fresh and healthy Arabidopsis leaves. Before inoculation, we rinsed the leaf surface with distilled water and wiped the leaf surface dry. The diameter of the lesion was measured. The Arabidopsis plants were monitored at 12 h intervals post-inoculation, and photographs were taken 36 h later.

### 2.8. Evaluation of Resistance of Transgenic Plants to B. cinerea

Following the manufacturer’s guidelines, the levels of malondialdehyde (MDA), proline, peroxidase (POD), superoxide dismutase (SOD), and catalase (CAT) activities were assessed using a plant detection kit (Solarbio, Beijing, China). Briefly, 0.1 g of plant sample was added to 1 mL of extraction liquid, homogenized, and then centrifuged; the supernatant was collected and finally determined by using a spectrophotometer. The levels of superoxide radicals (O_2_^−^) and hydrogen peroxide (H_2_O_2_) in the leaves were assessed through nitro blue tetrazolium (NBT) [32] and 3, 3′-diaminobenzidine (DAB) staining [33].The leaf samples were vacuum-infiltrated with NBT solution, which reacts with superoxide anion radicals to form a dark blue formazan compound that is insoluble. After dyeing, the leaves were soaked in 70% ethanol (*v*/*v*) until all chlorophyll was completely removed. Another portion of the sample was immersed in DAB solution and stained for 12 h in the dark. Following this, these samples were also soaked in 70% ethanol (*v*/*v*) until all chlorophyll was entirely removed.

### 2.9. Statistical Analyses

The experiments in this study were replicated thrice. Data analysis was carried out using Excel 2021 (Microsoft, Redmond, WA, USA). The outcomes are presented as the mean ± standard deviation (SD). Statistical analysis was performed utilizing SPSS Statistics 26.0 software (SPSS Inc., Chicago, IL, USA), and graphical representations were generated using GraphPad Prism 10 software (GraphPad software Inc., La Jolla, CA, USA).

## 3. Results

### 3.1. Phylogenetic Analysis of MnCYP710A11 Gene

**Figure 1:**
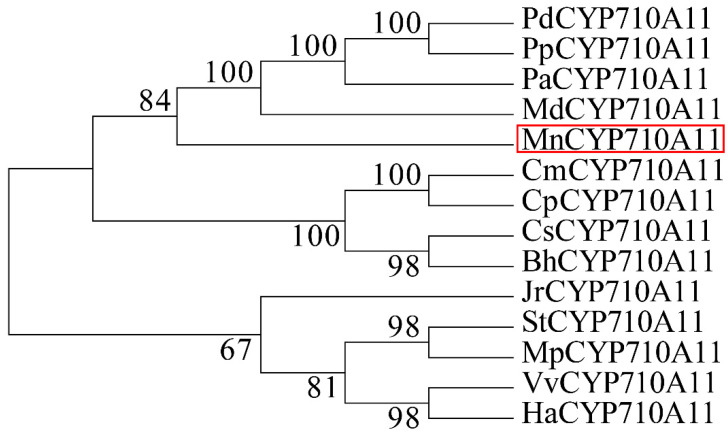
A phylogenetic tree of CYP710A11 proteins in mulberry and other plants. The number represents the confidence percentage. The accession numbers for these protein sequences obtained from GenBank are as follows: *PdCYP710A11* (*Prunus dulcis*, XP_034213338.1); *PpCYP710A11* (*Prunus persica*, XP_007213284.2); *PaCYP710A11* (*Prunus avium*, XP_021805687.1); *MdCYP710A11* (*M. domestica*, XP_008371595.2); *CmCYP710A11* (*Cucurbita moschata*, XP_022926360.1); *CpCYP710A11* (*Cucurbita pepo*, XP_023518762.1); *CsCYP710A11* (*Cucumis sativus*, XP_004134602.1); *BhCYP710A11* (*Benincasa hispida*, XP_038883734.1); *JrCYP710A11* (*Juglans regia*, XP_018859536.2); *StCYP710A11* (*Senna tora*, KAF7840462.1); *MpCYP710A11* (*Mucuna pruriens*, RDX60276.1); *VvCYP710A11* (*V. vinifera*, RVW16710.1); and *HaCYP710A11* (*H. annuus*, XP_022035515.1).

Multiple alignment was performed with other plant CYP710A11 protein sequences obtained from the NCBI database. Subsequently, phylogenetic and molecular evolutionary analyses were conducted utilizing MEGA 7 to investigate the evolutionary connections among various species (Figure 1). The results indicated that CYP710A11 in mulberry was closely related to CYP710A11 in *Malus domestica* and more distantly related to CYP710A11 in *Vitis vinifera* and *Helianthus annuus*.

### 3.2. B. cinerea-Induced MnCYP710A11 Expression

**Figure 2:**
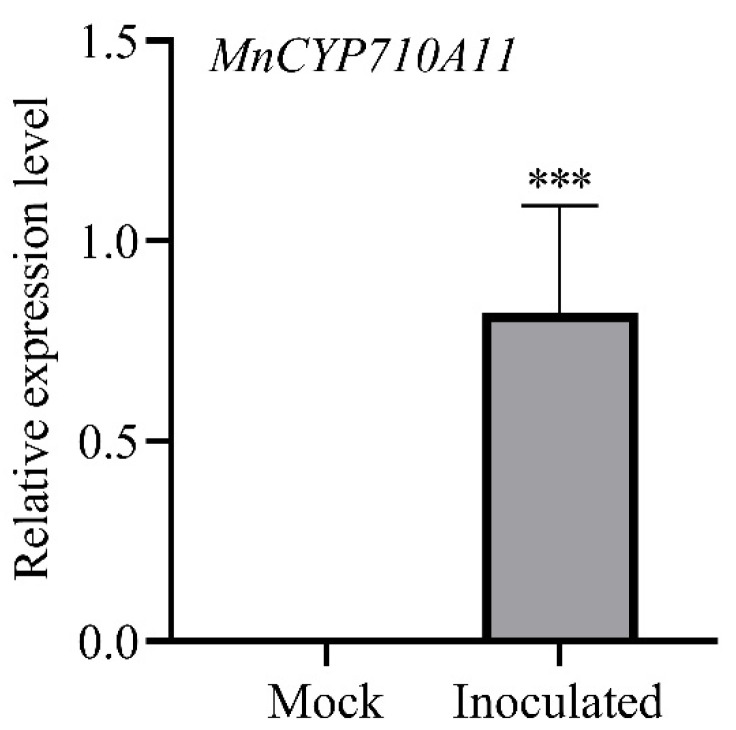
The expression levels of *MnCYP710A11* in mulberry leaves following mock treatment (Mock) and after inoculation with *B. cinerea* (Inoculated) was compared. The values represent averages, and the standard error (SE) is depicted as an error bar, based on three independent biological samples, each with three technical replicates (*** *p* < 0.001; two-tailed *t*-test). Mock, inoculated *B. cinerea*-free agar blocks; Inoculated, inoculated agar blocks containing *B. cinerea*.

The expression level of *MnCYP710A11* in mulberry seedlings infected with *B. cinerea* was examined using qRT-PCR analysis (Figure 2). Compared with 3 days of mock treatment, the expression level of *MnCYP710A11* was significantly increased after 3 days of inoculation, consistent with our previous transcriptome findings [24]. This suggests that the *MnCYP710A11* gene could play a role in enhancing mulberry’s resistance to *B. cinerea*.

### 3.3. Ectopic Expression of MnCYP710A11

**Figure 3:**
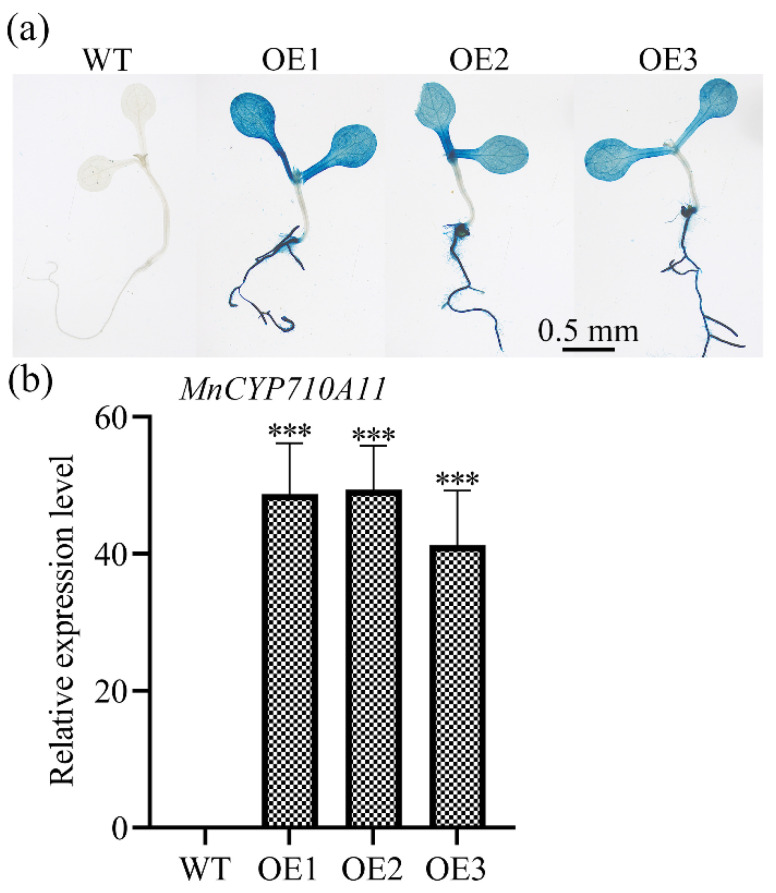
The identification of transgenic Arabidopsis. (**a**) Transgenic Arabidopsis GUS staining. (**b**) An assessment of the relative expression levels of *MnCYP710A11* in transgenic Arabidopsis. WT, wild Arabidopsis; OE, *MnCYP710A11* transgene Arabidopsis. The values represent averages, and the standard error (SE) is depicted as an error bar, based on three independent biological samples, each with three technical replicates (*** *p* < 0.001; two-tailed *t*-test).

To further validate the role of the *MnCYP710A11* gene in resistance, we introduced the *MnCYP710A11* gene into Arabidopsis for heterologous expression. The cDNA of *MnCYP710A11* was incorporated into Arabidopsis under the regulation of the *Cauliflower mosaic virus* 35S promoter, leading to the acquisition of T3 transgenic Arabidopsis plants following screening. First, the positive transgenic plants were verified by GUS staining. Following GUS staining, the transgenic plants exhibited a blue coloration (Figure 3a). Subsequent validation through qRT-PCR confirmed the overexpression of the *MnCYP710A11* gene (Figure 3b). These outcomes affirm the successful overexpression of the *MnCYP710A11* gene in Arabidopsis, resulting in the generation of positive transgenic plants.

### 3.4. MnCYP710A11 Transgenic Plant Enhances Resistance to B. cinerea

**Figure 4:**
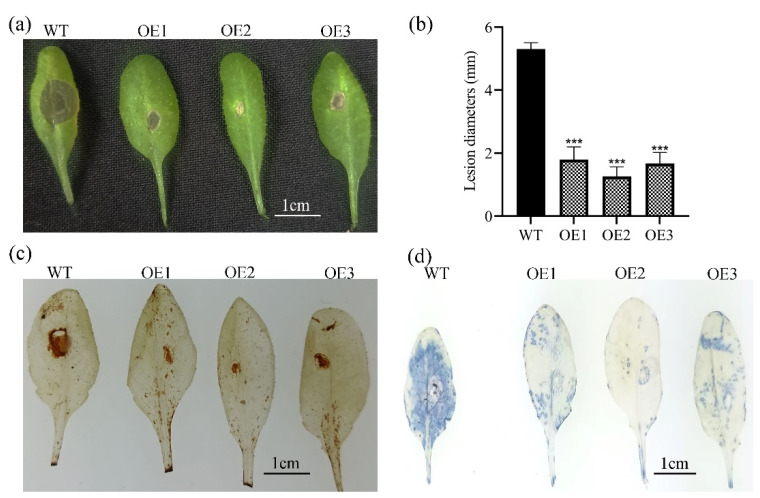
Evaluation of transgenic Arabidopsis resistance to *B. cinerea*. (**a**) Observation of leaf morphology in Arabidopsis 36 h post-infection with *B. cinerea*. (**b**) Quantitative assessment of resistance in transgenic Arabidopsis. (**c**) DAB staining showed H_2_O_2_ levels. (**d**) NBT staining showed O_2_^−^ levels. Values represent averages, and standard error (SE) is depicted as error bar, based on three independent biological samples, each with three technical replicates (*** *p* < 0.001; two-tailed *t*-test).

To evaluate the resistance of Arabidopsis transgenic plants overexpressing *MnCYP710A11* against *B. cinerea*, we conducted an experiment where transgenic Arabidopsis leaves were inoculated with agar blocks containing *B. cinerea* hyphae (Figure 4a). After 36 h of inoculation, while control leaves exhibited severe lesions, the leaves of *MnCYP710A11* overexpression lines only showed mild lesions. Quantitative analysis clearly demonstrated that the overexpression of *MnCYP710A11* in Arabidopsis effectively suppressed the infection induced by *B. cinerea* (Figure 4b). In addition, the production of reactive oxygen species represents a plant’s reaction to stress. To determine this, we conducted DAB staining and NBT staining to detect the levels of hydrogen peroxide (H_2_O_2_) and superoxide (O_2_^−^) in the leaves, respectively (Figure 4c,d). The results showed that Arabidopsis plants transfected with *MnCYP710A11* displayed minimal dark-brown patches after DAB staining, indicating lower levels of H_2_O_2_ accumulation. Conversely, the WT Arabidopsis plants exhibited large dark-brown patches, suggesting higher H_2_O_2_ accumulation. Similarly, NBT staining revealed minimal dark-blue patches in Arabidopsis plants transfected with *MnCYP710A11*, indicating lower levels of O_2_^−^ accumulation, whereas large dark-blue patches were observed in the WT *Arabidopsis* plants, indicating higher O_2_^−^ accumulation.

### 3.5. Detection of Biochemical Indices

**Figure 5:**
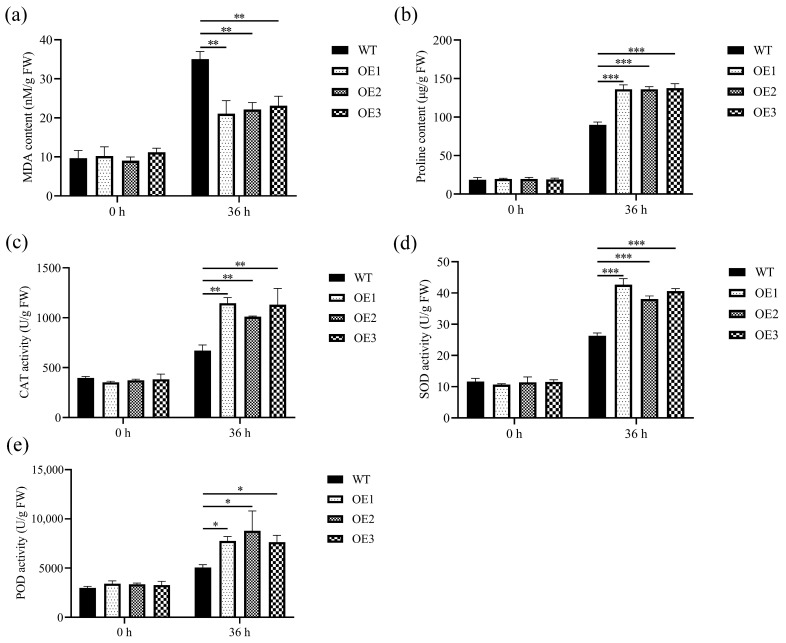
The determined physicochemical indexes before and after *B. cinerea* inoculation. (**a**) MDA content, (**b**) proline content, (**c**) CAT activity, (**d**) SOD activity, and (**e**) POD activity. The values represent averages, and the standard error (SE) is depicted as an error bar, based on three independent biological samples, each with three technical replicates (* *p* < 0.05, ** *p* < 0.01, and *** *p* < 0.001; two-tailed *t*-test).

We assessed the variations in malondialdehyde (MDA) content under both normal conditions and during *B. cinerea* infection to assess cell membrane damage (Figure 5a). The findings revealed no notable disparity in MDA content between wild-type (WT) and transgenic Arabidopsis plants during normal growth conditions. However, the MDA content of WT and transgenic Arabidopsis increased 36 h after infection by *B. cinerea* compared with 0 h. Meanwhile, the MDA content of transgenic plants was significantly lower than that of WT plants after 36 h of infection with *B. cinerea*. These results indicate that plasma membrane damage is more pronounced in WT plants than in transgenic Arabidopsis.

During normal conditions, there was no substantial variance in proline content between wild-type (WT) Arabidopsis and those overexpressing *MnCYP710A11* (Figure 5b). The proline content of wild-type and transgenic plants increased 36 h after *B. cinerea* infection compared with 0 h. At the same time, the proline content of transgenic Arabidopsis was significantly higher than that of wild-type plants 36 h after *B. cinerea* infection.

Catalase (CAT), superoxide dismutase (SOD), and peroxidase (POD) are crucial enzymes in plants that are pivotal in eliminating reactive oxygen species (ROS). During normal conditions, there were no notable variances in the activities of CAT, SOD, and POD between wild-type (WT) and transgenic Arabidopsis leaves (Figure 5c–e). However, the CAT, SOD, and POD activities of transgenic Arabidopsis were significantly increased compared with those of WT plants 36 h after infection with *B. cinerea*.

### 3.6. Disease Resistance Analysis of Mulberry Seedlings Overexpressing MnCYP710A11

**Figure 6:**
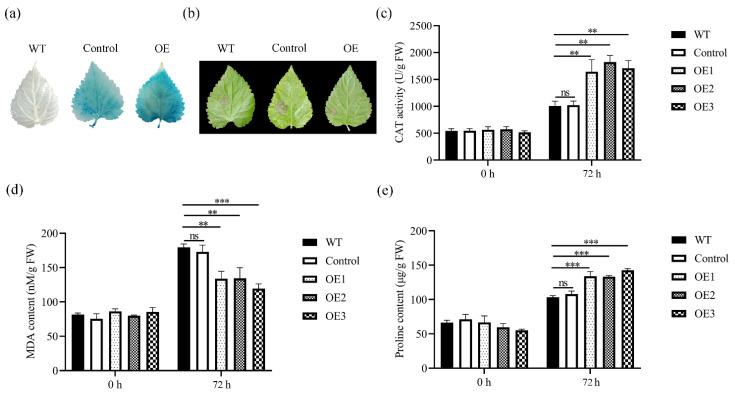
The resistance of *MnCYP710A11* to *B. cinerea* was analyzed through transient expression in mulberry. (**a**) GUS staining of WT plants and transient overexpression after 72 h in mulberry leaves. WT, wild-type; Control, empty vector. (**b**) The photos were taken 72 h after infection with *B. cinerea* of mulberry leaves. (**c**) MDA content, (**d**) proline content, and (**e**) CAT activity. The values are averages and the standard error (SE) is represented by a bar representing three independent biological samples with three technical replicates per sample (** *p* < 0.01 and *** *p* < 0.001; two-tailed *t*-test). ns, no significant difference.

For a more in-depth exploration of *MnCYP710A11*’s role in mulberry trees, a transient overexpression of *MnCYP710A11* was induced in mulberry trees (Figure 6). Histochemical examination of β-glucuronidase (GUS) displayed intense GUS staining in the leaves of mulberry seedlings, confirming the successful overexpression of *MnCYP710A11* in mulberry trees (Figure 6a). Compared with WT plants, mulberry trees overexpressing *MnCYP710A11* showed increased resistance to *B. cinerea* (Figure 6b). Upon *B. cinerea* infection, the transient expression of *MnCYP710A11* led to a notable reduction in malondialdehyde (MDA) content in mulberry seedlings (Figure 6c), while simultaneously enhancing proline content and catalase (CAT) activity (Figure 6d,e). These findings align with the earlier results observed in *MnCYP710A11* transgenic Arabidopsis.

### 3.7. Enhancement of AtBG2 Expression in MnCYP710A11 Transgenic Plants

**Figure 7:**
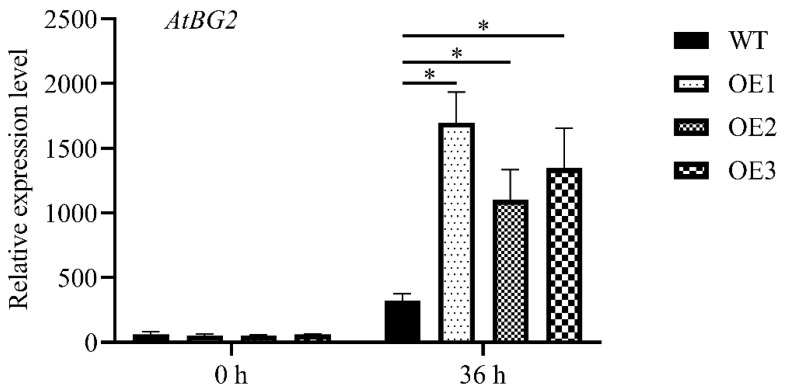
The relative expression of *AtBG2* in leaves of Arabidopsis WT and *MnCYP710A11* transgenic (OE) before and after inoculation with *B. cinereal*. The values are averages and the standard error (SE) is represented by a bar representing three independent biological samples with three technical replicates per sample (* *p* < 0.05; two-tailed *t*-test).

β-1,3-glucanase 2 (BG2) serves as a marker gene associated with plant defense mechanisms. The findings revealed no notable variance in *AtBG2* gene expression between transgenic Arabidopsis plants transfected with *MnCYP710A11* and wild-type (WT) plants prior to *B. cinerea* infection (see Figure 7). However, compared with 0 h, *AtBG2* expression levels in transgenic Arabidopsis plants transfected with *MnCYP710A11* and WT plants were upregulated 36 h after infection with *B. cinerea*. At the same time, the expression level of transgenic plants was significantly higher than that of WT plants 36 h after infection with *B. cinerea*. These findings indicate that the introduction of the *MnCYP710A11* gene into Arabidopsis can induce the expression of resistance-related genes to combat *B. cinerea* infection.

## 4. Discussion

The identification of plant resistance genes is the basis of breeding resistant varieties. Transcriptomic analysis has been utilized to identify genes that govern plant resistance against pathogen infections [34,35]. In our prior research, we conducted comparative transcriptomic analyses of mulberry trees after *B. cinerea* infection and obtained candidate genes that may regulate *B. cinerea* resistance. As a reactive enzyme, cytochrome P450 is extensively distributed among animals, plants, and microorganisms. Certain P450 enzymes have been documented to catalyze the production of diverse primary and secondary metabolites in plants. However, the roles of most cytochrome P450 genes in mulberry remain unknown. In this study, we chose the CYP450 protein-coding gene CYP710A11 to validate its role in disease resistance, thereby enhancing the precision of our findings.

Within plants, the CYP450 protein is categorized into 10 distinct clans spanning 61 families [36]. In this study, we isolated and characterized a CYP450 gene, CYP710A11, from mulberry, classified under the CYP710 subfamily. The CYP710 family is widely distributed in plants, exhibiting diverse structures and functions, with its members playing a role in sterol biosynthesis [14,37,38]. For example, overexpression of the *WsCYP710A11* gene in transgenic hairy roots of *Withania* resulted in a significant elevation of withanolides and phytosterol levels [38]. Although the role of CYP710 in sterol biosynthesis is well established, there is limited information on the disease resistance mechanisms of the CYP710 subfamily in plants. This study demonstrated that the overexpression of CYP710A11 improved the resistance of Arabidopsis and mulberry against *B. cinerea*. These findings offer fresh perspectives on the role of the P450 710 subfamily.

Reactive oxygen species (ROS) and ROS enzymes are crucial factors in enhancing plant resistance against *B. cinerea*. For example, ABA can reduce resistance to *B. cinerea* in tomatoes by reducing NO production, which also inhibits ROS and ethylene production [39]. In addition, the disruption of cuticle integrity mediated by peroxide-dependent ROS accumulation plays an important role in the strong resistance of plants with altered homogalacturonan integrity to *B. cinerea* [40]. ROS can harm cellular constituents such as lipids, proteins, and nucleic acids, leading to elevated levels of MDA, which serves as a marker for lipid peroxidation. [41]. When faced with oxidative stress, plants trigger the activation of antioxidant enzymes like superoxide dismutase (SOD), catalase (CAT), and peroxidase (POD) to eliminate reactive oxygen species (ROS) [42]. Plants additionally amass osmolytes like proline to uphold osmotic equilibrium and safeguard cellular structures against dehydration [43]. In this study, in comparison to the WT, the parameters of Pro, SOD, CAT, and POD were significantly increased after infection with the gray mold, while the parameter of MDA was significantly decreased after the overexpression of CYP710A11. This indicates that the overexpression of *MnCYP710A11* helps prevent cell membrane damage and enhances the capacity to preserve cell integrity in response to *B. cinerea* infection. Proline accumulation leads to hypersensitivity in incompatible interactions between plants and pathogens. At the same time, proline catabolism can act as a regulatory center of defense-related metabolism in many eukaryotes, connecting metabolic activities of different subcellular compartments and promoting and integrating ROS signals to regulate pathogen response [44]. The overexpression of *MnCYP710A11* may increase plant hypersensitivity by increasing proline content and may combine with ROS signaling pathways to enhance resistance against *B. cinerea*.

The level of callose (β-1,3-glucan) is controlled through the coordinated action of callose synthetase (CalSs) and β-1,3-glucanase [45,46]. The deposition of callose between the plasma membrane (PM) and the cell wall serves as a complex defense mechanism exhibited by the plant host when encountering pathogen infection [47], for instance, stimulated by MAMP activation (such as bacterial flagellate epitopes like flg22 and chitosan), as well as by filamentous fungal attacks and physical injury [48,49,50]. This callose accumulation helps fight diseases, such as fungal infections [51]. The presence of β-1,3-glucanase in *V. vinifera* had an inhibitory effect on *Plasmopara viticola* [52]. In our study, the BG2 gene associated with callose biosynthesis was upregulated in CYP710A11-OE plants, suggesting that this pathway is activated in CYP710A11-OE plants.

## 5. Conclusions

Our findings indicate that the CYP450 protein-coding gene CYP710A11 actively enhances the immune response in mulberry, thereby broadening our comprehension of the potential role of P450 proteins and offering valuable insights into the molecular mechanisms of plant immunity. Additionally, following the overexpression of *MnCYP710A11*, the resistance of mulberry to *B. cinerea* was augmented, thereby presenting significant genetic resources for the breeding of disease-resistant mulberry varieties.

## Data Availability

All of the data supporting the results of this study can be found online and in the Supplementary Materials.

## Supplementary Materials

The following supporting information can be downloaded at: https://www.mdpi.com/article/10.3390/genes15070853/s1, Table S1: Primers for real-time PCR.
