# Supplementary material for: Potential Involvement of MnCYP710A11 in Botrytis cinerea Resistance in Arabidopsis thaliana and Morus notabilis
Source: Genes (Basel). 2024 Jun 28;15(7):853. doi: 10.3390/genes15070853 (PMC11275358; doi:10.3390/genes15070853)
Supplement: Supplementary file 1 [file genes-15-00853-s001.zip › genes-3066918-supplementary.pdf]

Table S1. Primers for real-time PCR.

Gene symbol	Forward primer	Reverse primer
AtBG2	TAAGAGCTTTCTCGAACCAG	AGTACCCTGGATCGTTATCA
MnCYP710A11	CGCAGACTATGTTATCGGAA	TCCGGTCATGTAGATGAGAT
MnActin	GCATGAAGATCAAGGTGGTG	CATCTGCTGGAAGGTGCTAA
AtActin	TGCTGAGCTTATCGATTCCG	TTCGGTGATGGGAATACAG
